# Population dynamics with a mixed type of sexual and asexual reproduction in a fluctuating environment

**DOI:** 10.1186/1471-2148-12-49

**Published:** 2012-04-10

**Authors:** Roberto Barbuti, Selma Mautner, Giorgio Carnevale, Paolo Milazzo, Aureliano Rama, Christian Sturmbauer

**Affiliations:** 1 Dipartimento di Informatica, Università di Pisa, Pisa, Italy; 2 Institut für Zoologie, Karl-Franzens Universität, Graz, Austria; 3 Dipartimento di Scienze della Terra, Università degli Studi di Torino, Torino, Italy

## Abstract

**Background:**

*Carassius gibelio*, a cyprinid fish from Eurasia, has the ability to reproduce both sexually and asexually. This fish is also known as an invasive species which colonized almost all continental Europe, most likely originating from Asia and Eastern Europe. Populations of both sexually and asexually reproducing individuals exist in sympatry. In this study we try to elucidate the advantages of such a mixed type of reproduction. We investigate the dynamics of two sympatric populations with sexual and asexual reproduction in a periodically fluctuating environment. We define an individual-based computational model in which genotypes are represented by *L *loci, and the environment is composed of *L *resources for which the two populations compete.

**Results:**

Our model demonstrates advantageous population dynamics where the optimal percentage of asexual reproduction depends on selection strength, on the number of selected loci and on the timescale of environmental fluctuations. We show that the sexual reproduction is necessary for "generating" fit genotypes, while the asexual reproduction is suitable for "amplifying" them. The simulations show that the optimal percentage of asexual reproduction increases with the length of the environment stability period and decrease with the strength of the selection and the number of loci.

**Conclusions:**

In this paper we addressed the advantages of a mixed type of sexual and asexual reproduction in a changing environment and explored the idea that a species that is able to adapt itself to environmental fluctuation can easily colonize a new habitat. Our results could provide a possible explanation for the rapid and efficient invasion of species with a variable ratio of sexual and asexual reproduction such as *Carassius gibelio*.

## Background

There is an ongoing discussion about the maintenance of sexual reproduction in most eukaryotes despite its evolutionary costs: recombination can break up favourable sets of genes accumulated by selection and asexual populations comprising only females can reproduce twice as fast in each generation than bisexual populations without the need to produce males for ongoing reproduction [1]. Despite these considerable evolutionary costs, sexual reproduction is still by far the most frequent mode of reproduction found in vertebrates [2] whereas asexual reproduction has only been described in less than 0.1% of vertebrate species [3].

Various patterns exist for the coexistence of sexual and asexual species. Many non-vertebrates are not limited to either sexual or asexual reproduction. Species often live or even coexist in sexual and asexual lineages, either alternating throughout their life-cycle [4] or in spatially or temporally isolated populations [5]. Exclusively unisexual vertebrates are usually closely related to sexually reproducing species [6,7] with many examples demonstrating coexistence of unisexual and bisexual populations [8-11]. Several hypotheses have been presented in order to explain the absence of the twofold advantage [12,13] many of them based on the discrimination ability and the efficiency of males during mate choice of sexual reproduction [14-17].

Unisexual lineages can evolve by various mechanisms (spontaneous, contagious or infectious origin, hybridization) from ancestral sexual species [18]. Most unisexually reproducing species have been described to originate from one or multiple hybridization events involving bisexual species [19,20]. Most unisexual/bisexual complexes appear to be of paraphyletic origin, some cases of a polyphyletic relationship between unisexual and bisexual lineages are known, indicating multiple hybridization events [21].

*Carassius gibelio *or Gibel carp, a cyprinid fish from Eurasia, is so far the only vertebrate species described in which sexually and asexually reproducing natural populations coexist sympatrically [22-27]. In unisexual natural populations *Carassius gibelio *individuals are all females with a triploid genome and reproduce asexually by a mechanism called gynogenesis (sperm-dependent parthenogenesis) [28]. During gynogenetic reproduction, offspring are parthenogenetically formed but egg development cannot be completed without sperm. In general, male DNA is not incorporated into the offspring's genome but triploid gynogenetic females are dependent on sperm donors [9] which usually belong to a closely related species. In bisexual populations both gynogenetic and gonochoristic types of reproduction coexist. When the eggs of a female are inseminated with sperm from other species, the paternal genome makes no contribution to the offspring.

However, when the eggs are inseminated with homologous sperm from silver crucian carp males, the entered sperm decondenses and becomes a male pronucleus, and then the male pronucleus undergoes fusion with the female pronucleus [22,24]. Although some authors suggest a hybridization event of *Cyprinus carpio *and *Carassius auratus *[29,30] to explain the origin of asexual Gibel carp, *Carassius gibelio *has also been described as the only known vertebrate species with a variable ratio of sexual and asexual reproduction of non-hybrid origin [9].

In general, it is assumed that sexually reproducing populations contain more genetic variation than asexually reproducing populations, and that a high level of genetic variation allows perpetual adaptation to changing environments [31,32]. Particularly populations in heterogeneous habitats threatened by various parasites or under strong competition have been shown to contain a high level of genetic variation [20,33]. Genetic variation results from both mutation and recombination where mutation generates new alleles in both sexual and asexual species, and recombination in vertebrates only occurs in sexually reproducing species during meiotic division. Rearrangement of alleles during recombination not only increases genetic variation but also protects against the effects of Muller's ratchet [34,35]. In asexual species new mutations are inherited by the offspring without any changes facilitating an accumulation of deleterious mutations [36]. While sexual populations show a greater diversity of multilocus genotypes, unisexual populations can display a greater divergence between alleles within one locus because the two copies will accumulate different mutations over time [37,38]. Furthermore, increased levels of allelic diversity and heterozygosity at different loci have been found in some unisexual populations [39,40].

Depending on the ecological conditions in a habitat, bisexual populations might replace faster reproducing unisexual lineages because of the long-term benefits of increased genetic variation [32]. Due to the long-term advantages of sexual reproduction, asexually reproducing species were classically considered as evolutionary dead-ends until the discovery of "ancient asexuals" [37,38,41,42]. Their existence demonstrates asexuality as stable evolutionary status under particular circumstances. However, most asexual species indeed represent phylogenetically young groups [43].

In this paper we study, by a computational model, the advantages of a mixed type of sexual and asexual reproduction in a changing environment following the idea that a species that is able to adapt itself to environmental fluctuation can easily colonize a new habitat and that a particular ratio of sexual versus asexual reproduction is favoured by certain levels of environmental stability. Our results could provide a possible explanation for successful invasion of species such as *Carassius gibelio *which are able to colonize new regions very quickly and efficiently.

## Methods

### The model

To study the advantages of a mixed type of sexual and asexual reproduction we model two abstract fish populations, each with a certain percentage of the population reproducing asexually and both populations competing for the same resources. This situation is unlikely to occur in nature where asexuality is rare but it offers a way of understanding the power of asexual reproduction in adapting to changing or new environments.

Each population is characterized by a certain percentage of asexual and sexual reproduction. The percentage of asexual reproduction ranges between 0% (complete sexual reproduction) and 100% (all the females reproduce by gynogenesis). To study the behaviour of the two populations with respect to the resources they compete for, we assume that they exploit sperm from a third species and that sperm is not limiting.

We consider diploid individuals whose genotype is represented by *L *loci, each one assuming alleles in {0, 1}. Because of the diploid assumption we have 2^2*L *^possible genotypes, ranging from $\underset{2L}{\underset{︸}{00\ldots 00}}$ to $\underset{2L}{\underset{︸}{11\ldots 11}}$. Thus a genotype g is represented by a sequence of *L *pairs $\text{g}=\left\langle \left({l^{\prime }}_{1},{l^{\prime \prime }}_{1}\right),\left({l^{\prime }}_{2},{l^{\prime \prime }}_{2}\right)\ldots ,\left({l^{\prime }}_{L},{l^{\prime \prime }}_{L}\right)\right\rangle$, where each pair $\left({l^{\prime }}_{i},{l^{\prime \prime }}_{i}\right)$ represents the alleles at locus *i*. We can think to these *L *loci as the ones which mainly control the fitness of individual for the environment, where each locus corresponds to the fitness for an ecological trait.

The environment is represented by *L *resources, *env *= ⟨*r*_1_, *r*_2_,..., *r_L_*⟩, each one with values in {0, 1}. The fitness of a genotype $\text{g}=\left\langle \left({l^{\prime }}_{1},{l^{\prime \prime }}_{1}\right),\left({l^{\prime }}_{2},{l^{\prime \prime }}_{2}\right)\ldots ,\left({l^{\prime }}_{L},{l^{\prime \prime }}_{L}\right)\right\rangle$ is computed, with respect to the environment *env*, as follows:

$$F\left(\text{g},env\right)=e^{-\;\;\left(\frac{{\left(1\;-\;lf\right)}^{2}}{2\sigma^{2}}\right)}$$

where *σ *is a parameter measuring the strength of the ecological selection (smaller values of *σ *correspond to a stronger selection), and *lf*, the *loci fitness*, based on the multiplication of the fitness of each locus, is defined as:

$$\text{lf}=\overset{L}{\underset{i=1}{\prod }}f\left(\left({l^{\prime }}_{i},{l^{\prime \prime }}_{i}\right),r_{i}\right)$$

Function *f *is defined as follows:

$$f\left(\left({l^{\prime }}_{i},{l^{\prime \prime }}_{i}\right),r_{i}\right)=\left\{\begin{array}{cc} 1 & \text{if}{l^{\prime }}_{i}={l^{\prime \prime }}_{i}=r_{i}\\ 1-\delta s & \text{if}{l^{\prime }}_{i}\neq {l^{\prime \prime }}_{i}\\ 1-s & \text{if}{l^{\prime }}_{i}={l^{\prime \prime }}_{i}\neq r_{i} \end{array}\right)$$

where *s *is the fitness decrement at a single locus and *δ *is a dominance factor between alleles.

The function *f *measures the fitness of the ecological trait represented by one locus to the corresponding feature of the environment. The loci fitness function *lf *considers the multiplicative effect of each ecological trait to the individual fitness. Finally, the fitness function ℱ is a exponential function in which the slope depends on the strength of the selection *σ*. ℱ is equal to 1 if all the ecological traits (represented by loci) are fit with respect to the environment, and it decreases exponentially following the multiplicative effect of unfit loci. A similar function is adopted in [44,45]. Let us remark that, in this paper, we consider *δ *= 0.5, that is each allele contributes equally to the fitness of a locus. In [45], where a dominance factor among alleles is considered, *δ *can assume different values. In computing the fitness of individuals, each locus contributes independently from the others. Results can be different if some form of epistasis is considered [46].

We assume that the population has one reproductive season each year. During this season all females reproduce. Each female has the possibility to reproduce in either sexual or asexual mode with a probability which is proportional to the percentages of sexual/asexual reproduction in her population. If the chosen form of reproduction is asexual, the female produces all females offspring copying her own genotype. In sexual mode a male is chosen at random and the offspring are probabilistically composed by half males and half females with genotypes obtained by recombination of the parental alleles. In this process each locus segregates independently.

The reproductive season is followed by a viability selection. During this phase the probability that an individual of genotype *g *survives in an environment *env, p_surv_*(g, *env*), is given by a slight modification of the Beverton-Holt model [47-51]:

$$p_{surv}\left(\text{g},env\right)=\frac{1}{1+b\;\phi \;\frac{N}{\text{K}\left(\text{g},env\right)}}$$

where *b *is the average number of offspring per female which can reach the free-swimming stage in the environment, *ϕ *is the percentage of females in the population, *N *is the number of adults in the population, and K(g, *env*) is the carrying capacity associated with the genotype g in *env*. K(g, *env*) is given by K(g, *env*) K_0 _where K_0 _is the maximum carrying capacity of the environment. The modification of the standard Beverton-Holt model is motivated by the fact that we consider overlapping generations and that we apply the viability selection, based on survival probability, not only to young fishes but to all individuals in the population. Notice that in our model, in which two populations compete for the same resources, *N *is the total number of individuals in the two populations.

We assume that the young fishes are able to reproduce at the age of one year. Thus, newly born fish are able to have offspring in the next reproductive season. We define the maximum carrying capacity to be 30,000 individuals. For a population the initial sex ratio depends on the percentage of sexual/asexual reproduction in the population itself. In particular if a population has a percentage *α_sex _*of sexual reproduction, and a percentage *α*_asex _= 1-*α*_sex _of asexual reproduction, its initial population is composed of a percentage of $\alpha_{\text{asex}}+\;\frac{\text{1}}{2}\alpha_{\text{asex}}$ females, and a percentage of $\frac{\text{1}}{2}\alpha_{\text{sex}}$ males.

In this paper, we do not deal with the reasons for a coexistence of sexual and asexual populations. To allow such a coexistence, in the simulated populations we eliminate the twofold advantage of asexual reproduction by increasing the strength of selection for the asexually produced offspring. In particular, in the presence of a population *p *with percentages *α*_sex _and *α*_asex _of sexual and asexual reproduction, respectively, we set the strength of selection, *σ*_p_, for the population as follows:

$$\sigma_{\text{p}}=\sigma \alpha_{\text{sex}}+\mu \sigma \alpha_{\text{asex}}$$

where *μ*, 0 *< μ ≤ *1, is the factor of the selection increase for all individuals produced asexually. In the following we set *μ *= 0.2.

Note that assigning the same selection strength for sexually and asexually generated individuals means to leave unmodified the "cost of sex". Such a cost will create a strong disadvantage for the population with a greater percentage (*α*_sex_) of sexual reproduction, thus it will be outcompeted by the other population in every environment condition. Because sexual/asexual complexes exist in natural systems, we know that there are natural mechanisms for eliminating the advantage of asexual propagation. We consider that our way of eliminating the twofold advantage of asexual reproduction is a simplification of natural mechanisms. Some studies show that male mate choice can contribute to the stability of sexual/asexual complexes [11,16]. In this case females of the asexual populations suffers from the fact that males can disregard them. Only fit females (the ones which can attract males) can reproduce. In our model we allow all the females to reproduce, but we reduce the offspring number by applying to a part of the population (corresponding to the percentage of asexual reproduction) a stronger selection. We think that this method is general enough for approximating different mechanisms underlying the stability of sexual/asexual complexes.

The value 0.2 for *μ *corresponds to setting the parameter *σ*, measuring the strength of the selection against asexually produced fishes, five times the one for sexually reproduced ones.

### Modelling strategy

We perform simulations by considering initial populations with different percentages of asexual reproduction. The percentages start from 0% (complete sexual reproduction) and, by a step of 10, reach 100% (complete asexual reproduction). For each combination of percentages in the two populations we perform 5 simulations, thus, because pairs of different percentages occur twice in this process, for each combination (excluded the ones in which the percentages in the two populations are equal) we perform 10 simulations. Each initial population comprises 9,000 individuals. Given the carrying capacity of the environment, the total population rapidly reaches nearly 30,000 fish.

We consider three different values of *L*, the number of loci: 3, 5 and 7. Initially, both populations have an intermediate fitness where all individuals are heterozygous at each locus. This kind of initial population is considered only for simplicity in simulations. We obtain the same results starting from two populations with the same fitness and in which the alleles for each locus are present at the same frequency.

With different numbers of loci we use different values of *s *in order to have individual loci fitness values inside the interval [~0.5, 1]. To obtain these values we use *s *= 0.2 when *L *= 3, *s *= 0.125 when *L *= 5 and *s *= 0.09 when *L *= 7.

Every simulation runs for 500 generations. The final result can be either of the following three: a) population 1 survives and population 2 becomes extinct, b) both populations survive, and c) population 1 becomes extinct and population 2 survives. Actually, when selection is strong both populations can go towards extinction but this case is not interesting for our purpose.

A *stability period, π*, of the environment is considered, in which the environment does not change. At the end of such a stability period a fluctuation occurs, the environment changes completely, that is each one of the L features composing the environment is complemented. Thus, if *env *= ⟨*r*_1_, *r*_2_,..., *r_L_*⟩, the new environment is given by *env' *= ⟨1-*r*_1_, 1-*r*_2_,..., 1-*r_L_*⟩. In the simulations we consider three different fluctuating environments with stability periods of 50, 20 and 10, respectively.

During sexual and asexual reproduction we allow mutations which alter the value of an allele of a new individual. Mutations are recurrent [52] and do not produce new alleles but only change a 0 allele into a 1 allele and vice versa. The allele and the locus to be altered are randomly chosen. We use a mutation rate of 10^-5^. Because we consider a number of offspring reaching the first year, before the viability selection, equal to 10, a mutation rate equal to 10^-5 ^correspond to 3 allelic mutations every generation.

Two different levels of ecological selections are taken into account, which correspond to different values of *σ*: weak selection (*σ *= 0.7) and strong selection (*σ *= 0.5).

### Implementation

The simulations were performed using Open Watcom C for Windows [53]. For *L *= 3 loci a single simulation for 500 generations takes nearly 65 seconds, for *L *= 5 nearly 120 seconds and for *L *= 7 nearly 300 seconds. The code is available on request.

## Results

Simulation results show that the optimal percentage of asexual reproduction varies with all of the parameters we have considered: the number of loci^a ^in the genotype *L*, the strength of the ecological selection *σ *and the length of the stability period *π*. In particular, the optimal percentage of asexual reproduction increases with longer stability periods and decreases with higher values of *L *and *σ*.

### Direct comparison of populations with different rates of asexuality

**Figure 1:**
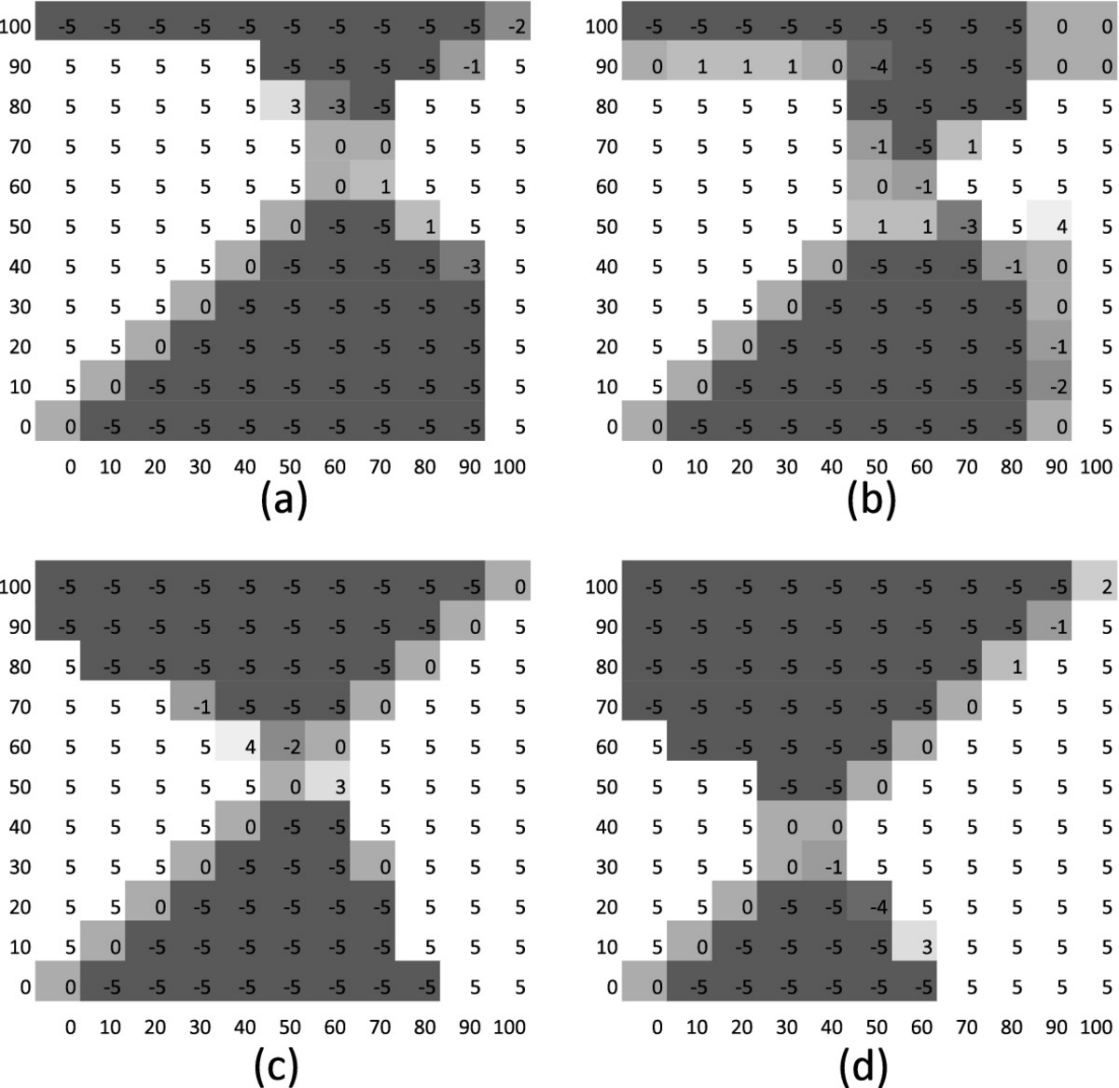
**Comparison between two diploid populations (with *L *= 3 loci) with different percentage of asexual reproduction in a fluctuating environment**. The two populations are in sympatry and compete for the same resources but they do not interbreed. Population 1 is on the vertical axis while population 2 is on the horizontal axis. In each scheme the percentage of asexual reproduction varies between 0 and 100 in step of 10 and each crossing is colored in relation with which population survived best: white is where population 1 prevailed, dark where population 2 did, and tones of grey in the middle. The four schemes are expression of different selection strength (columns, from weakest on the left to strongest on the right) and of different environment fluctuation periods (rows, longer on the top and shorter on the bottom). Details of subfigures: (a) *σ *= 0.7 and *π *= 50; (b) *σ *= 0.5 and *π *= 50; (c) *σ *= 0.7 and *π *= 10; (d) *σ *= 0.5 and *π *= 10.

The simulations performed a comparison between two diploid populations (with *L *= 3, *L *= 5 and *L *= 7 loci) with different percentages of asexual reproduction in a fluctuating environment. The two population occur in sympatry and compete for the same resources but they do not interbreed. The outcome of the simulation series, for *L *= 3, is summarized in Figure 1. Population 1 is shown on the vertical axis while population 2 is shown on the horizontal axis. In each of the four schemes the percentage of asexual reproduction varies between 0% and 100% in steps of 10% and each crossing of the schemes is coloured in relation to which population survived best: white is where population 1 prevailed, dark where population 2 did, and tones of grey if survival was in the middle. The schemes in the left column had weak selection (*σ *= 0.7) while the right column had strong selection (*σ *= 0.5). The upper row was in a slowly changing environment (*π *= 50) and the lower row in a quickly changing environment (*π *= 10).

In case where the stability period of the environment is equal to 50 generations we observe that high percentages of asexual reproduction enables a population to adapt well to the ecological features of the environment. In fact, given the long period of environment stability, a population can exploit the small percentage of sexual reproduction for generating the "good" genotypes, and it can profitably use the high percentage of asexual reproduction for "amplifying" them. When the percentage of asexual reproduction reaches 100%, the advantage of recombination is lost. In this case the generation of "good" genotypes relies on mutations only. Because the mutation rate is low, such a population cannot compete with populations with a percentage of sexual reproduction. On the other hand, a completely sexual population (0% of asexual reproduction) cannot compete, in most cases, with the efficiency of the asexual population in multiplying well adapted genotypes.

Figure 1 shows that, when the stability period is shortened and the selection is strengthened the populations have an advantage by a higher level of sexual reproduction. Thus, while a stability period of 50 generations has the greatest advantage with 60*-*70% of asexual reproduction, for a period of 10 generations and strong selection the best percentages of asexual reproduction are 30*-*40%.

This might be explained by the fact that this percentage of sexual reproduction is necessary to react efficiently to environmental changes, and sexual reproduction must be occur often enough to allow the population to produce a good percentage of fit genotypes in a time shorter than the stability period.

The results of the simulations with *L *= 5 and *L *= 7 are not shown. They essentially confirm the results obtained with *L *= 3 with a slower dynamics. Intuitively, adaptation by sexual reproduction takes more time with more complex genotypes.

### Diffusion of fit genotypes in the general population

The simulations show other interesting details about how fast different percentages of asexuality influence population demography. In particular, we focused on the percentage of fit individuals in the population. To study the adaptation phase we define a *fit genotype *as the one having a loci fitness greater than or equal to 0.8, *lf ≥ *0.8. We considered a single population composed of 28,000 individuals without any fit genotype, and we ran some simulation for 100 generations. For each simulation we recorded the dynamic of fit genotypes with respect to the whole population.

**Figure 2:**
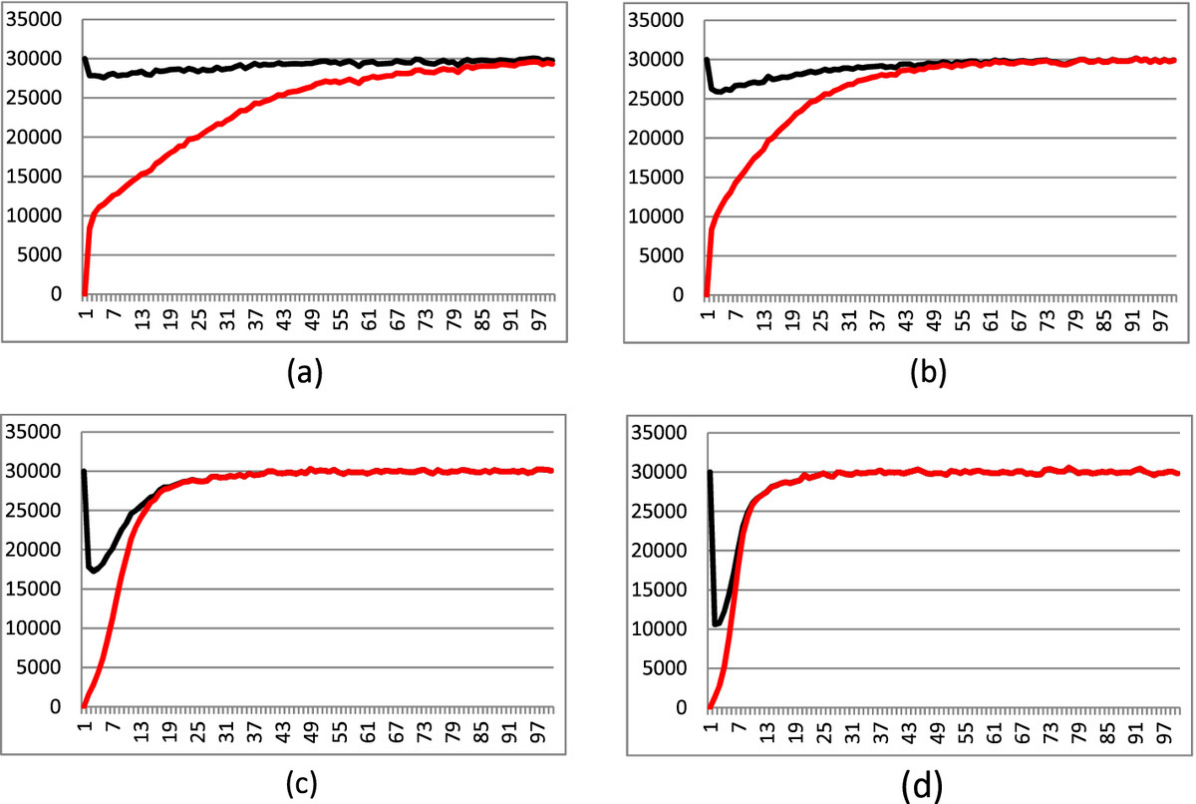
**Adaptation phases of a diploid population (with *L *= 3 loci)**. The curves represent the total population (top) and the part of the population with fit genotypes (individuals with *lf ≥ *0.8). The vertical axes report the number of individual and the horizontal ones the number of generations. The initial populations has no fit genotype. The four graphs are expression of different selection strength (columns, from weakest on the left to strongest on the right) and of different percentage of asexual reproduction (rows, 0% asexual on top, 80% asexual on bottom). Top lines give the total amount of individuals in the population, while the bottom ones give the number of fit genotypes. The columns of the figures correspond to values of *σ *equal to 0.7 and 0.5, from left to right. Details of subfigures: (a) *σ *= 0.7 and 0% asexual; (b) *σ *= 0.5 and 0% asexual; (c) *σ *= 0.7 and 80% asexual; (d) *σ *= 0.5 and 80% asexual.

The results show that the time to reach a percentage of 100% fit genotypes decreases both with the strength of selection and with the percentage of asexual reproduction. It is important to notice an interesting behaviour: the way in which the percentage of 100% of fit genotypes was reached differed greatly with the differences in percentages of asexuality. Essentially there were two phases in the adaptation process. A first phase in which fit genotypes must be created (*generation phase*). This phase was better achieved by sexual populations, thus the generation phase was faster when asexuality decreased. A second phase in which fit genotypes must be multiplied (*amplification phase*). The amplification phase was better performed when asexuality increased. These results, for *L *= 3, are summarized in Figure 2.

We do not show the results with *L *= 5 and *L *= 7 which are coherent with the ones for *L *= 3. As expected when the number of loci increase the adaptation phase takes more time to complete.

### Diffusion of fit genotypes with different rates of asexuality

**Figure 3:**
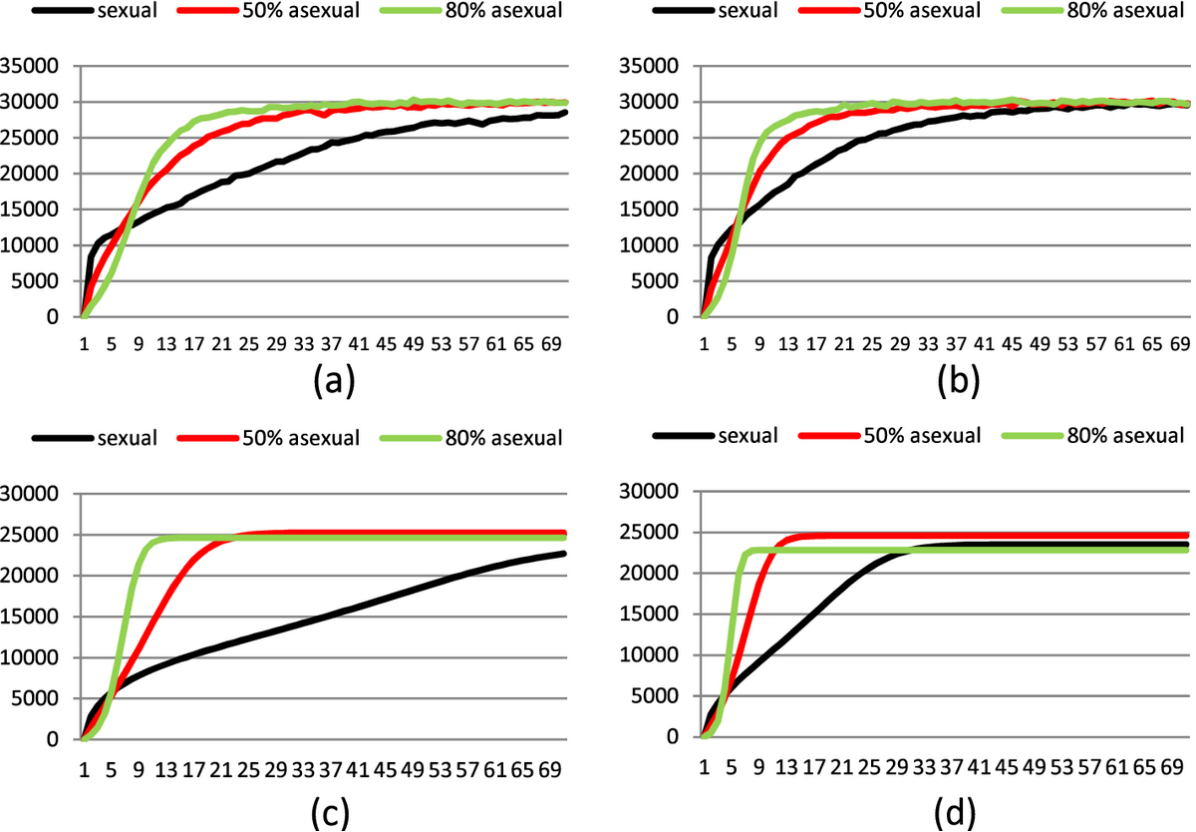
**Amount of fit genotypes with different percentage of asexual reproduction (0%, 50% and 80%) in a diploid population (with *L *= 3 loci) as computed by the stochastic simulation (top row) or by the deterministic functions (bottom row)**. The number of fit genotypes is on the vertical axes and the number of generations on the horizontal one. Graphs (a) and (c) are expression of a weakest selection (*σ *= 0.7) while graphs (b) and (d) of a stronger selection (*σ *= 0.5).

As noted before, the speed of diffusion of fit genotypes in the population changed with the rate of asexuality. We analyze the adaptation phases of three populations (completely sexual, 50% asexual reproduction, 80% asexual reproduction) with *L *= 3, taking into consideration the number of generations needed to reach 100% of fit genotypes, starting from a population composed by only unfit genotypes. The time for adaptation is longer when the sexuality in a population increases and when the selection decreases. These results are summarized in Figure 3.

To appreciate the initial growths, the graphs show only 70 generations. The generation phase and the amplification phase can be roughly distinguished in the graphs. With complete sexuality the generation phase is quite short, while the amplification phase is long. When the percentage of asexual reproduction grows, the generation phase slows but the amplification phase becomes faster. Recall that all the populations have an initial composition of unfit genotypes only. This is not the situation in a standard simulation because when an environment fluctuation occurs the stabilization phase does not start necessarily with all unfit genotypes. Thus the growth curves of fit genotypes in Figures 2 cannot be used as absolute values for explaining the results of simulations, but they give the adaptation trends for populations.

### Population size in environments with different stability periods

**Figure 4:**
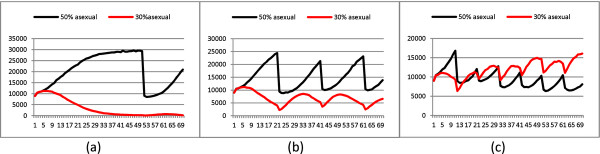
**First 70 generations of two populations**. First 70 generations of two populations in presence of strong selection (*σ *= 0.5). The compared populations have: 30% of asexual reproduction (red line) and 50% of asexual reproduction (black line). The three graphs refer to periods, *π *of 50, 20 and 10 generations, from left to right respectively.

To explain better the last point we analyze the consistency of two populations with different percentages of sexuality and we consider different environment stability periods. We see that a long stability period, *π *= 50, allows the population with 50% of asexuality to outcompete the other one by completing both the generation and amplification phases before the environment fluctuation. The environment fluctuation provokes a great decline in the population without really menacing it. A shorter period, *π *= 20, do not allow the 50% population to completely perform the amplification phase, thus endangering its survival and allowing the other population to perform better and survive. Finally a very short period, *π *= 10, allows the population with only 30% asexuality to exploit its greater speed in the generation phase before the environmental fluctuation. In this case the population with a higher rate of sexual reproduction was at an advantage. These results are summarized in Figure 4.

It is important to remark that the value *μ *= 0.2 allows us to have clear results of the simulations. By increasing the value of *μ*, that is by decreasing the selection on asexually reproduced individuals, we can get analogous results of simulations by shortening the environment stability periods. This would make the population fluctuations faster, thus decreasing the readability of results. On the other hand, decreasing the value of *μ *should give a greater advantage to sexually reproduced individuals, which could outcompete asexually reproduced ones, independently from the environment changes. Because we want to study the influence of environment fluctuations on the populations with respect to their kind of reproduction, we found that *μ *= 0.2 gives understandable results.

### A deterministic model

From the simulations we can infer an approximated deterministic model which allows us to reason on the relations among the parameters, as well as give some previsions on the success of a population in a fluctuating environment. Consider a population *N^t^*, of *N *individuals at generation *t*, split in those with a fit genotype, $N_{f}^{t}$, and those with an unfit one, $N_{u}^{t}$. Consider percentages, *α_sex _*and *α_asex_*, of sexual and asexual reproduction in the population. For simplicity we use a model without overlapping generations. The dynamics of the two parts of the population, $N_{f}^{t}$ and $N_{u}^{t}$, can be approximated by the following equations:

$$\begin{array}{c} {\text{N}}_{\text{f}}^{\text{t}+1}=\;\;\left(\alpha_{\text{asex}}\;b\;\phi \;N_{\text{ f}}^{\text{t}}\right)\\ \;\;\;\;\;\;\;\;\;\;\;\;\;\;\;\;+\;\;\alpha_{\text{asex}}\;b\;\phi \;N_{\text{f}}^{\text{t}}\left(1-\delta_{\text{fu}}\left(1-\frac{N_{\text{f}}^{\text{t}}}{N}\right)\right)\\ \;\;\;\;\;\;\;\;\;\;\;\;\;\;\;\;-\;\;\alpha_{\text{asex}}\;b\;\phi \;N_{\text{f}}^{\text{t}}\delta_{\text{fu}}\left(1-\frac{N_{\text{f}}^{\text{t}}}{N^{\text{t}}}\right)\\ \;\;\;\;\;\;\;\;\;\;\;\;\;\;\;\;+\;\;\alpha_{\text{asex}}\;b\;\phi \;N_{\text{f}}^{\text{t}}\left(\delta_{\text{uf}}\right)\frac{1}{1+\left(b-1\right)\;\phi \;\frac{N^{\text{t}}}{{\overset{-}{\text{K}}}_{\text{f}}}}\\ N_{u}^{t+1}=\;\left(\alpha_{\text{asex}}\;b\;\phi \;N_{\text{u}}^{\text{t}}\right)\\ \;\;\;\;\;\;\;\;\;\;\;\;\;\;\;+\;\;\alpha_{asex}\;b\;\phi \;N_{\text{u}}^{\text{t}}\left(1-\delta_{\text{uf}}\right)\\ \;\;\;\;\;\;\;\;\;\;\;\;\;\;\;-\;\;\alpha_{\text{asex}}\;b\;\phi \;N_{\text{u}}^{\text{t}}\delta_{\text{uf}}\\ \;\;\;\;\;\;\;\;\;\;\;\;\;\;\;+\;\left(\;\alpha_{\text{asex}}\;b\;\phi \;N_{\text{f}}^{\text{t}}\delta_{\text{fu}}\left(1-\frac{N_{\text{f}}^{\text{t}}}{N^{\text{t}}}\right)\right)\frac{1}{1+\left(b-1\right)\;\phi \;\frac{N^{\text{t}}}{{\overset{-}{\text{K}}}_{\text{u}}}} \end{array}$$

where *b *is the average number of offspring for female, *ϕ *is the percentage of females in the population (based on the percentages of sexual and asexual reproduction, as explained in Section), *δ_fu _*is the approximate percentage of unfit genotypes produced by fit genotypes, while *δ_uf _*is the approximate percentage of fit genotypes produced by unfit ones. Remark that, with strong approximation, we consider that random mating do not alter the percentage of the two parts of the populations, thus we consider only the changes due to fit parents producing unfit offspring, and to unfit parents producing fit offspring. Of course *δ_fu _*and *δ_uf _*are estimated a priori considering the possible recombination of all fit genotypes as well as the possible recombination among the unfit ones. The factor $\left(\text{1}-\frac{N_{f}^{t}}{N^{t}}\right)$ expresses the fact that when the whole population is composed of fit genotypes the number of unfit alleles in the population is so small that the generation of unfit genotypes is negligible. Dynamically the composition, in the evolving population, of fit and unfit genotypes can change significantly, thus *δ_fu _*and *δ_uf_*, estimated as before, can result in inaccurate values. $N_{f}^{t}$ and $N_{u}^{t}$ produce clones at *α*_asex _b *ϕ *rate. Finally, the term $\frac{1}{1+\left(\text{b}-1\right)\;\phi \frac{{\text{N}}^{\text{t}}}{{\overset{-}{\text{K}}}_{\text{u}}}\;}$ represent the selection according to the Beverton-Holt model. ${\overset{-}{\text{K}}}_{\text{f}}$ and ${\overset{-}{\text{K}}}_{\text{u}}$ are estimates of the average carrying capacity of fit and unfit genotypes, ${\overset{-}{\text{K}}}_{\text{f}}={\overset{-}{F}}_{\text{f}}{\text{K}}_{0}$ and ${\overset{-}{\text{K}}}_{\text{u}}=\;{\overset{-}{F}}_{\text{u}}{\text{K}}_{0}$, where ${\overset{-}{F}}_{f}$ and ${\overset{-}{F}}_{u}$ are the average fitness of fit and unfit genotypes. In order to have readable results we set $\delta_{fu}=\;\;0.\text{25}\left(\text{1}-\frac{\text{1}}{L}\right)$ and $\delta_{uf}=0.0\text{21}+0.\text{28}\frac{1}{L}$. With these values we obtain, for *L *= 3, the curves given in the graphs (c) and (d) of Figure 3 (which approximate the stochastic simulations represented in the same figure by graphs (a) and (b)). These curves do not overlap the ones obtained by the simulations, however, they show the same trend with respect to the parameters *σ *and *α*. In the top graphs of the figure, the final consistencies of the three populations slightly differ given the non precise effect of the population carrying capacity computed as average of the ones of all genotypes.

## Discussion

Stable coexistence of asexual sperm parasites and their sexual host species seems paradoxical. Any all-female asexual species should replace its sexual host because sexual females must bear the cost of producing males unless sexual and asexual females do not compete for the same ecological niche. Nevertheless there are many examples of stable sexual/asexual complexes (a general discussion about the mechanisms for the stability of these complexes can be found in [9,11,12]). In a recent study of the ovoviviparous and gynogenetic Amazon molly (*Poecilia formosa*) and its sexual host species (*Poecilia latipinna*) [16], the authors found that a higher percentage of *P. latipinna *had sperm in their genital tract than *P. formosa*. Moreover, in all the observed conditions, *P. latipinna *always had a greater amount of sperm. These results are consistent with male mate choice contributing to the stability of this sexual/asexual complex. Moore and McKay [8] presented a model describing the interaction among sexual and asexual populations belonging to the genus *Poeciliopsis*. This model was based on a parameter controlling the strength of male mate choice and another one measuring the increase of the insemination of asexual females due to social interactions among males. More recently, Heubel et al. [14] elaborated a model in which male mate preferences and male efficiency (essentially the number of females which can be fertilized by a male) are considered. Coexistence of sexual and asexual populations depends on the fact that sperm becomes more limiting for gynogens when they reach a high density. The model of coexistence provided by Mee and Otto [17] is based on a polymorphism occurring in the sexual host species. Sexual females have two genotypes *E*_1 _and *E*_2_. *E_i _*males prefer to mate with sexual females by a factor *a_i_*, thus *E*_i _males mate with sexual females *a_i _*times more frequently than with asexual females.

By tuning the parameters, the dynamic of the model approaches, by oscillations, a stable equilibrium where gynogens, *E*_1 _and *E*_2 _are present. However, for Crucian carp *Carassius auratus*, male mate choice does not strongly motivate the existence of sexual/asexual complexes [15], as the involved species spawn openly in usually conspecific groups. Olofsson and Lundberg [13] studied the dynamics of sexual/asexual complexes by a mathematical model. Their model considers fixed density selection strength for the populations without adaptation to the environment.

Many authors consider that reduced heterozygosity, due to asexuality, negatively influences population fitness. In particular reduced heterogeneity in a population can cause an increased vulnerability to parasites as described in the Red Queen hypothesis [54-61].

In this paper, we do not try to explain the coexistence of sexual and asexual populations, but we investigate the advantage of a mixed type of reproduction in a single species when colonizing new environments. We compare different populations and, supported by the example described above, remove the two-fold advantage of gynogenesis, that is the doubled amount of individuals who are capable of producing offspring that are born in an female-only asexual population with respect to a sexual population of the same size [52]. In the model we eliminate the twofold advantage of asexual reproduction by a simplification of natural mechanisms. We allow all the females to reproduce but we reduce the number of asexually produced offspring by applying to a part of the population (corresponding to the percentage of asexual reproduction) a stronger selection.

The idea of this study comes from the existence of a so far unique vertebrate *Carassius gibelio*, which is able to reproduce both gynogenetically and sexually. Moreover, *Carassius gibelio *is known for its strong ability to adapt to new environments. This ability allowed rapid colonization of almost all freshwater of continental Europe, most likely originating from Asia and Eastern Europe. Actually, it is not known which is the percentages of sexual and asexual reproduction in wild populations, although it could be inferred by the frequency of males in such populations. In [22] a percentage of males of 20% in some natural populations is reported, which should correspond to a percentage of 40% of sexual reproduction. Apart from real data, the aim of this paper was to study the advantages given by combining the two modes of reproduction when the environment is unstable. The results we have obtained cannot be used as quantitative, depending on parameters whose values are unknown for natural environment, but they can explain qualitatively the dynamics of populations when the values of the parameters change. In particular, a greater percentage of sexual reproduction is advantageous either when the environment fluctuations are frequent or when the selection is strong, but it is disadvantageous either in a stable environment or in presence of mild selection. The contemporary presence of sexual and asexual reproduction has been seldom documented. Among vertebrates the unique known case, so far, is *Carassius gibelio *[26], while there are examples among invertebrates [62,63]. Much more widespread, among invertebrates, is cyclical parthenogenesis: phases of reproduction are alternate with phases of sexual one. Example of organisms adopting that mixed strategy of propagation are cladocerans, rotifers and aphids. *Daphnia *(Crustacea, Branchiopoda, Cladocera) is a cyclical parthenogen living in freshwater. Under favourable conditions *Daphnia *reproduces by parthenogenesis, producing clones which constitute a population of all females. This can take place for one to several generations. When the environment conditions are unfavourable (presence of predators, food shortage, cold temperature) the females switch to sexual reproduction [64-66]. Males are generated parthenogenetically and females start to produce haploid eggs which must be fertilized. When a fertilized egg is encapsulated in an ephippium, a chitinous membrane, it can become a dormant egg that undergoes a diapause to survive the winter season The relative percentages of parthenogenetic and sexual generations depend on the conditions of the environment.

Although our model consider sexual and asexual reproduction occurring at the same time, when the selection is strong, it can model with sufficient accuracy cyclical parthenogenesis. With strong selection unfit genotypes are quickly removed, either when they are generated by the hatching of dormant eggs (like in *Daphnia*) or when they are produced during the reproduction season (like in *Carassius gibelio*). Thus, when the selection is strong, the only difference between cyclical parthenogenesis and contemporary sexual and asexual propagation is the time in which new genotypes are generated. Moreover, an environment fluctuation in our model is similar to a winter season for *Daphnia*. This because, with strong selection, an environment change causes a big decline in the population consistency. Thus, our model can approximate the behaviour of a population with cyclical parthenogenesis by representing the length of the parthenogenetic phase with the percentage of asexual reproduction, and the diapause periods with environment fluctuations.

From the simulations we performed we can infer the following assumptions explaining the adaptability of a species with two modes of reproduction (sexual/asexual). First of all we show that a population using sexual reproduction only produces different genotypes very quickly. This is due to recombination and segregation with their maximal expression in pure sexual reproduction. The occurrence of new genotypes is delayed by introducing a certain percentage of asexual reproduction. If selection is present, unfit genotypes suffer a disadvantage, its magnitude depending on the strength of selection, and tend to be eliminated. Balloux et al. [39] showed that genotype diversity in a population will be maintained if the population itself retains a percentage, although very small, of sexual reproduction. This is not in contrast with our results because [39] did not consider selection, giving all genotypes the same survival probability, and genotype heterogeneity is maintained despite a high percentage of asexual reproduction.

The combined effect of asexual reproduction (delaying the effect of recombination) and selection is well represented in Figure 3 showing the time for producing fit genotypes and the generation and amplification phases. We can note that higher percentages of asexual reproduction^b ^allow the population to quickly acquire fit genotypes. Thus, we conclude that, under the assumption of our model, in a stable environment any population with a higher percentage of asexual reproduction has an advantage with respect to the other populations. This does not mean that the population will ever push the other populations to extinction because initial conditions can alter this outcome (for example if the consistency of one population is significantly higher).

Our results are compatible with those of Misevic et al. [67], in which the digital organisms of the Avida system are used. Such organisms can reproduce either sexually or asexually. Reported experiments show that sexual reproduction can be predominant only in a rapidly changing environment. Keightley and Otto [68] showed that a small percentage of sexual reproduction (0.01 and 0.05) can be an advantage in the presence of deleterious mutations even by taking into account the costs of sexual reproduction. In particular, they quantify the costs of sexual reproduction by limiting the number of offspring. In the presence of a great number of deleterious mutations (which to some extent can be seen as changes in the stability of the environment) a certain percentage of sexual reproduction has a positive effect.

## Conclusions

In this paper we use an individual based computational model for studying the dynamics of populations with a mixed type of reproduction, sexual and asexual, in a fluctuating environment. To this purpose we simulated the evolution of two virtual populations, each one with a different percentage of asexual reproduction, competing for the same resources.

We show that the sexual reproduction is necessary for "generating" fit genotypes, while the asexual reproduction is suitable for "amplifying" them. Moreover, from the simulations, we can conclude that the optimal percentage of asexual reproduction increases with the length of the environment stability period and decreases with the strength of the selection and the number of loci.

Our results could provide a possible explanation for the rapid and efficient invasion of species with a variable ratio of sexual and asexual reproduction such as *Carassius gibelio*.

## Endnotes

^a^All the figures show results for *L *= 3 loci only, for brevity: while we ran simulation for *L *= 5 and *L *= 7 loci, the results are analogous and only changed in the speed of adaptation and mutation.

^b^However, the percentage of asexual reproduction should be less than 100% because with complete asexual reproduction the generation of new genotypes relies on mutations only

## Authors' contributions

GC, SM, and CS provided the biological background and proposed the study. RB, PM and AR conceived the computational model and programmed the simulator. All authors contributed to writing the final version of the paper. All authors read and approved the final manuscript
